# Incidence and predictors of loss to follow-up among South Sudanese refugees with HIV receiving care in Adjumani District, Uganda

**DOI:** 10.1016/j.ijregi.2025.100653

**Published:** 2025-04-22

**Authors:** Christopher Nyolonga, Joshua Uchaki Ufoyrwoth, Trinity Wanok, David Komakech, Joseph Baruch Baluku, Felix Bongomin

**Affiliations:** 1 Department of Medical Microbiology and Immunology, Faculty of Medicine, Gulu University, Gulu, Uganda; 2 Division of Pulmonology, Directorate of Internal Medicine, Kiruddu National Referral Hospital, Kampala, Uganda; 3 Department of Internal Medicine, Gulu Regional Referral Hospital, Gulu, Uganda

**Keywords:** HIV, Loss to follow-up, Refugees, Living with HIV

## Abstract

**Objectives:**

HIV care pose a significant challenge in refugee population. We determined the incidence and predictors of loss to follow-up (LTFU) among South Sudanese refugees with HIV in Uganda.

**Methods:**

We conducted a retrospective cohort study in public health facilities in Adjumani District, Uganda, involving South Sudanese refugees with HIV. LTFU was defined as missing 3 or more consecutive months at any point in their care since the start of the study period. A Cox proportional hazards regression analysis was fitted to determine independent predictors of LTFU and results are presented as adjusted hazard ratios (aHRs) with corresponding 95% confidence intervals (CIs).

**Results:**

We included data of 449 participants, with a median age of 37 (interquartile range: 30-43) years. Most were female patients (75.5%, n = 339) receiving care from health center (HC) III (87.3%, n = 392), with baseline World Health Organization stage 1 (79.3%, n = 341), negative baseline tuberculosis status (89.1%, n = 302), and suppressed viral load (86.5%, n = 360) and had no co-morbidity (91%, n = 402). Overall, 25 (5.6%) were lost to follow-up. Factors independently associated with LTFU were being male (aHR: 2.6, 95% CI: 1.1-6.1, *P* = 0.03) and receiving care from HC IV (aHR: 3.0, 95% CI: 1.0-8.8, *P* = 0.04).

**Conclusions:**

LTFU among South Sudanese refugees with HIV in Uganda is substantial. Routine use of patient address locator forms, and improved quality of HIV care at HC IV and men-targeted outreaches and community involvement may reduce LTFU among refugees living with HIV.

## Highlights

• There is about a 6% loss to follow-up rate (LTFU) in South Sudanese refugees with HIV.
• Men and health center IV patients had higher LTFU risk.
• Targeted outreach and better HIV care may reduce LTFU.
• Findings inform refugee HIV care retention strategies.

## Introduction

Worldwide, HIV remains a major public health issue, with over 40.4 million (32.9-51.3 million) people living with HIV and there is ongoing transmission in all countries; however, some countries report an increasing trend in the number of infections [1]. In 2022, over 39 million people were estimated to be living with HIV and, of these, two-thirds are in the African region [1]. About 86% of people living with HIV knew their status, 76% were receiving antiretroviral therapy (ART), and 71% had suppressed viral loads [1]. HIV prevalence in Uganda is 5.8%, and this was higher in women (7.2%) than in men (4.3%) [2]. The annual incidence of HIV among adults (those aged 15 years and above) was 0.29%, and, in West Nile sub-region, HIV prevalence is at 2.8% [2]. The West Nile sub-region is in northwestern Uganda, forming part of the northern region. The Democratic Republic of Congo (DRC) borders it to the west, south Sudan to the north, and the Albert Nile to the East. Adjumani District is located on the eastern bank of the Albert Nile, although it is part of the West Nile sub-region, and hosts the largest number of refugees in Uganda 203671 refugees, primarily hosting South Sudanese refugees due to its proximity to south Sudan [3].

Uganda is the largest refugee host, providing refuge to over 1.5 million people as of early 2024. Of these, 24% are new arrivals from south Sudan and 13% from the DRC. Notably, registered Sudanese increased since the start of the Sudan conflict in April 2023, averaging more than 1990 per month over the last 3 months. Refugees from south Sudan still account for the largest number of refugees in Uganda (926,593) [4]. The prevalence of HIV among adults aged 15 years and older living in the Ugandan refuges settlements in 2021 was 1.5% and it was higher among women than men (1.8% vs 1.1%), and the prevalence in the West Nile Settlement is at 1.4% [5].

The United Nations Joint Program on HIV/AIDS initiated the 95-95-95 in June 2021 campaign to hasten the epidemic control of HIV [6]. One of its objectives was to achieve viral suppression in 95% of patients receiving ART by 2030. The other goals were to ensure that 95% of individuals with HIV are aware of their status and that 95% of those diagnosed with HIV infection receive sustained ART [6,7]. The 2016 World Health Organization (WHO) and Uganda Ministry of Health ART guidelines of test and treat (T&T) involves screening individuals for HIV infection and immediately initiating treatment for those who test positive. This approach aims to reduce disease progression and transmission [8]. Approximately 80.9% of people living with HIV are aware of their status and over 96.7% of these are on ART [2]. Although meeting the third United Nations Joint Program on HIV/AIDS targets necessitates prompt ART initiation, it is crucial for patients to remain engaged with the health care system for regular drug refills and monitoring tests. Reviews of ART programs in sub-Saharan Africa indicate that around 60-65% of patients were retained in HIV care after 2 to 3 years of ART initiation [6].

The 2018 Uganda HIV progress report highlighted suboptimal retention among ART-naive patients, with an approximately 20% loss to follow-up (LTFU) rate at 12 months [9]. Immediate initiation of ART after HIV diagnosis, as seen in T&T programs, may lead to patients not returning to HIV clinics, potentially negating the benefits of prompt treatment initiation [5,9,10]. A study in Masaka showed that 42.8% started ART within 7 days of diagnosis and over 15.6% of patients were lost to follow-up among those who began ART [9]. LTFU has been linked to drug resistance and poorer long-term treatment outcomes, including mortality [11].

In the era of T&T, it is imperative to have data on patient retention, particularly, in routine health care settings [12]. Retention in HIV care among refugee populations is a significant challenge; this study aimed to determine the incidence of LTFU and its associated factors among South Sudanese refugees with HIV attending public health facilities in Adjumani District, Uganda.

## Material and methods

### Study design

This was a retrospective cohort study conducted in the health facilities in Adjumani District, Uganda.

### Study setting

Adjumani District is bounded to the North by Moyo District, to the northeast by Obongi Madi Okolo, to the Northwest by Obongi District, to the southwest by Arua District, and to the northwest by Yumbe District. Adjumani, the district’s largest town, is roughly 125 km (78 miles) northeast of Arua, the sub-region’s largest city, via road. This place is roughly 436 km (271 miles) northwest of Kampala, Uganda’s capital and largest city, via road. The district’s coordinates are 03 23N, 31 47E or latitude 3.3845 and longitude 31.7820. It is divided into Adjumani East (which include the following sub-counties: Itirikwa sub-county, Arinyapi sub-county, Ofua sub-county, Dzaipi sub-county, Pakelle sub-county, and Pakelle town council) and Adjumani west (which include the following sub-counties: Adropi sub-county, Pachara sub-county, Adjumani town council, Ukusijioni sub-county, and Ciforo sub-county).

Refugee settlement in Adjumani houses over 235,698 refuges and 85% (n = 182,977) are women and children. Majority are from south Sudan, followed by Sudan and DRC [3]. These refugees are in different zones or level, which include Nyumamzi, Pagirinya, Ayilo, Maaji II, Ayilo II, Maaji III, Boroli I, Mirieyi, Alere, Agojo, Baratuku, Olua I, Mungula I, Boroli II, Olua II, and Mungula II. The district has many health centers (HCs), which include Mungula HC level IV, Pagirinya, Ayilo, Ayiri, Bira which is HC level III, Ayilo II, Pagirinya II, Maji C, Alere, Elema, and Agojo which is HC level II; these are the centers where the refugees received their HIV care services plus other health related services and from which data were collected [3,13].

### Study unit

Clinical notes of South Sudanese refugees living with HIV receiving care from the health facilities in Adjumani District, Uganda.

### Sample size calculation

Using the Kish Leslie formula, because the incidence of LTFU not known, we assumed incidence to be at 50 per 100 persons (50%), and, at a 5% significance, the minimum sample size was estimated to be 384. Assuming a conservative non-response rate of 10% (384/0.9), the adjusted minimum sample size was 427 participants. Therefore, we reviewed 449 eligible files.

### Sampling technique

We used stratified random sampling method, where the healthy centers were grouped into homogenous strata of HC IV and HC III and one HC II which provides HIV care services. HC III and IV provide HIV care services in Uganda; however, they differ in the structure. HC III serves at the sub-county level, offering outpatient services, maternity care, and a basic laboratory, staffed led by a senior clinical officer. HC IV, on the other hand, operates at the county level, providing all services of HC III but with additional inpatient wards, a surgical theater for emergency operations, and a senior medical officer alongside another doctor. It acts as a mini-hospital, bridging the gap between lower-level facilities and regional referral hospitals. From each stratum, a systematic sampling was used to get the required number of files for review from each stratum using the registers from the clinic as our sampling frame (the ART clinic register), we chose a random number from the registers and included every next third participant in the study. From each HC III, we sampled 100 files, making total of 300 files and 149 files from Mungula HC IV, totaling to the 449 files.

### Eligibility criteria

All files for South Sudanese refugees receiving care from the ART clinic between January 2023 and April 2024 in the selected HC III and IV were included. Files whose owners have been transferred out to received care from another health facilities were excluded.

### Study variable

#### Dependent variable

Our dependent variable was LTFU, which was defined as a patient who has not visited the health facility ART clinic in 3 or more consecutive months at any point in their care since the start of the study period [9].

#### Independent variables

The independent variables were socio-economic characteristics for the participants such as age, sex, level of education, employment status, address, viral suppression which was taken as viral load <200 copies per ml of blood or undetected, WHO clinical stage, co-morbidities such as those with HIV, and either one or more of chronic illness such as hypertension or diabetes mellitus or any other chronic disease.

#### Data collection tool

We used a predesigned questionnaire which captured the information from the patients’ files, such as date on initiation to ART, date of last Visit in the ART clinic, viral load, clusters of differentiation 4 count, and demographic characteristics of the patients. The questionnaire was pretested at Gulu Regional Referral Hospital to ensure it captured the necessary information for the study.

#### Data collection procedure

On every ART clinic day on each HC, we used the registers of patients as a sampling frame for the sampling. The sampled files were then put together and reviewed to gather the appropriate information using the pretested questionnaire.

#### Data quality control

The data from the files were extracted by trained research assistants and supervised by the principal investigator of the study to ensure accuracy.

#### Data management

Filled questionnaires were reviewed for completeness and then entered in Microsoft Excel 2023, where the data were cleaned and coded. The Excel workbook was password-protected to ensure confidentiality. The hard copy questionnaire was kept in a safe room by the principal investigator.

#### Data analysis plan

The coded data set was exported to STATA MP 18 for analysis. Numerical variables were summarized using median and interquartile range or mean and SD, depending on the normality of the numerical variables. Categorical variables were described using counts and corresponding relative frequencies. Normality was assed using the Shapiro–Wilk test.

### Incidence of loss to follow

LTFU was defined as a patient who has not visited the health facility ART clinic in 3 or more consecutive months at any point in their care during the study period between January 2023 and April 2024, and the incidence of LTFU was defined as the number of patients missing clinic visits for 3 or more consecutive months divided by the total follow-up time. This was summarized in relative frequency.

### Predictors of LTFU among participants

Statistical analyses were conducted to investigate factors associated with LTFU among study participants. Associations between LTFU and categorical independent variables were assessed using either the chi-square test or Fisher’s exact test, depending on the expected frequencies. For continuous variables, normality was evaluated; variables with a normal distribution were analyzed using the independent samples *t*-test, whereas non-normally distributed variables were assessed with the Mann–Whitney U test. Variables demonstrating a *P*-value less than 0.20 in univariate analyses were considered for inclusion in the multivariable Cox proportional hazards regression model to identify independent predictors of LTFU. The Cox model estimated adjusted hazard ratios (aHRs) with corresponding 95% confidence intervals (CIs). Statistical significance was set at *P* <0.05. The proportional hazards assumption was evaluated using Schoenfeld residuals, and model fit was assessed through the concordance index (C index).

### Ethical consideration

The study protocol was approved by the Gulu University Research Ethics Committee (GUREC-2024-818). The study was conducted according to the Declaration of Helsinki. The privacy and confidentiality of each participant was maintained throughout the different processes of participant enrollment into the study and data collection. During data presentation, only aggregated data, not individual information, were presented to maintain confidentiality.

## Results

### Baseline characteristics of the study participants

We enrolled 449 participants for the study, with a median age of 37 (interquartile range [IQR]: 30-43) years. Majority were female (75.5%, n = 339), in union (65.7%, n = 295), from HC III (87.3%, n = 392), with baseline WHO stage I (79.3%, n = 341), negative baseline tuberculosis status (89.1%, n = 302), and suppressed viral load (86.5%, n = 360) and had no co-morbidity (91%, n = 402). More than half who were enrolled at an ART clinic were diagnosed from the facility (59.7%, n = 268). A total of 183 (40.9%) had dolutegravir (DTG)-based baseline ART regimen; however, 97.1% (n = 435) are currently on DTG-based regimen. The median duration to another refill was 3 (IQR: 3-4) months. The baseline median weight and height were 54 (IQR: 48-60.1) kg and 167 (IQR: 159.2-171.2) cm, respectively. The median duration since diagnosed with HIV was 6 (IQR: 3-8) and initiated on ART was 6 (IQR: 3-8) years (Table 1).

**Table 1:** Baseline characteristics of the participants.

Variable	Frequency	Percentage
Age, median (IQR), years	37	30-43
Sex		
Female	339	75.5
Male	110	24.5
Marital status		
Not in union	154	34.3
In union	295	65.7
Baseline weight, median (IQR), kg (n = 397)	54	48-60.1
Baseline height, median (IQR), cm (n = 94)	167	159.2-171.2
Entry to ART clinic		
Diagnosed from facility	268	59.7
Transfer in	181	40.3
Level of facility		
2	21	4.7
3	392	87.3
4	36	8.0
Duration since diagnosed with HIV, median (IQR), years	6	3-8
Duration since initiation on ART, median (IQR), years	6	3-8
Baseline World Health Organization stage		
1	341	79.3
2	51	11.9
3	35	8.1
4	3	0.7
Baseline clusters of differentiation 4 count, median (IQR), counts	381	232-551
Baseline tuberculosis		
Negative	302	89.1
Positive	37	10.9
Baseline ART regimen		
DTG-based	183	40.9
LPVr/ATVr-based	4	0.9
NVP/EFV-based	261	58.2
Change regimen		
No	168	37.4
Yes	281	62.6
Current regimen		
DTG-based	435	97.1
LPVr/ATVr-based	2	0.5
NVP/EFV-based	11	2.4
Duration to refill, median (IQR), months	3	2-4
Viral load at 6 months, median (IQR), counts	400	151-883
Viral load suppression		
No	56	13.5
Yes	360	86.5
Co-morbidity		
No	402	91.0
Yes	40	9.0

ART, antiretroviral therapy; DTG, Dolutegravir; IQR, interquartile range; LPV/r, Lopinavir/ritonavir; ATV/r, Atazanavir/ritonavir; NVP, Nevirapine; EFV, Efavirenz.

### LTFU among south Sudanese refugees with HIV receiving care in Adjumani District, Uganda

The incidence of LTFU was 5.6 per 1000 participants (5.6%).

### Factors associated with LTFU among the participants

In the bivariate analysis, factors associated with LTFU among the participants were sex (*P* = 0.029), level of facility (*P* <0.01), duration since diagnosed with HIV (*P* = 0.005), duration since initiated on ART (*P* = 0.005), baseline ART regimen (*P* = 0.051), and duration to next refill (*P* = 0.025) (Table 2).

**Table 2:** Bivariate analysis for associated with LTFU among the participants.

Variable	LTFU		*P*-value
	No (n = 424) Frequency (%)	Yes (n = 25) Frequency (%)	
Age, median (IQR), years	37 (30-44)	35 (29-40)	0.373
Sex			
Female	325 (76.7)	14 (56.0)	0.029
Male	99 (23.4)	11 (44.0)	
Marital status			
Not in union	148 (34.9)	6 (24.0)	0.386
In union	276 (65.1)	19 (76.0)	
Entry to ART clinic			
Diagnosed from facility	251 (59.2)	17 (68.0)	0.411
Transfer in	173 (40.8)	8 (32.0)	
Level of facility			
2	20 (4.7)	1 (4.0)	0.010
3	374 (88.2)	18 (72.0)	
4	30 (7.1)	6 (24.0)	
Duration since diagnosed with HIV, median (IQR), years	6 (4-8)	3 (2-6)	0.005
Duration since initiation on ART, median (IQR), years	6 (3-8)	3 (2-6)	0.005
Baseline World Health Organization stage			
1	322 (79.3)	19 (79.2)	0.980
2	48 (11.8)	3 (12.5)	
3	33 (8.1)	2 (0.3)	
4	3 (0.7)	0 (0)	
Baseline tuberculosis			
Negative	284 (89.3)	18 (85.7)	0.489
Positive	34 (10.7)	3 (14.3)	
Baseline ART regimen			
DTG-based	167 (39.5)	16 (64.0)	0.051
LPVr/ATVr-based	4 (1.0)	0 (0)	
NVP/EFV-based	252 (59.6)	9 (36.0)	
Change regimen			
No	155 (36.6)	13 (52.0)	0.138
Yes	269 (63.4)	12 (48.0)	
Current regimen			
DTG-based	410 (96.9)	25 (100)	0.673
LPVr/ATVr-based	2 (0.5)	0 (0)	
NVP/EFV-based	11 (2.6)	0 (0)	
Duration to refill, median (IQR), months	3 (2-4)	3 (1-3)	0.025
Viral load suppression			
No	53 (13.2)	3 (23.1)	0.397
Yes	350 (86.8)	10 (76.9)	
Co-morbidity			
No	383 (90.8)	19 (95.0)	>0.999
Yes	39 (9.2)	1 (5.0)	

ART, antiretroviral therapy; DTG, Dolutegravir; IQR, interquartile range; LPV/r, lopinavir/ritonavir; ATV/r, Atazanvir/ritonavir; LTFU, loss to follow-up; NVP, Niverapine; EFV, Efavirenz.

### Factors associated with LTFU among south Sudanese refugees with HIV receiving care in Adjumani District, Uganda

In the simple Cox regression, factors associated with LTFU among the participants were being male (crude HR [cHR]: 2.6, 95% CI: 1.13-5.86, *P* = 0.024), receiving care from HC IV (cHR: 4.2, 95% CI: 1.53-11.25, *P* <0.01), on DTG-based baseline regimen (cHR: 2.7, 95% CI: 1.15-6.21, *P* = 0.021), Table 3. At multivariable analysis, factors independently associated with LTFU among the participants were being male was 2.6 times at risk of LTFU (aHR: 2.6, 95% CI: 1.11-6.10, *P* = 0.028) and receiving care from HC IV were three times at risk of LTFU (aHR: 3.01, 95% CI: 1.03-8.79, *P* = 0.044), (Table 3).

**Table 3:** Factors independently associated with loss to follow-up among the participants.

Variable	Crude HR (95% CI)	*P*-value	Adjusted HR (95% CI)	*P*-value
Sex				
Female	Reference		Reference	
Male	2.6 (1.13-5.86)	0.024	2.6 (1.11-6.10)	0.028
Level of facility				
2	1.04 (0.13-8.17)	0.971	1.05 (0.13-8.56)	0.967
3	Reference		Reference	
4	4.2 (1.53-11.25)	0.005	3.01 (1.03-8.79)	0.044
Baseline ART regimen				
DTG-based	2.7 (1.15-6.21)	0.021	1.5 (0.49-4.89)	0.457
LPVr/ATVr-based	—		—-	
NVP/EFV-based	Reference		Reference	
Duration to refill	0.75 (0.57-0.98)	0.039	0.81 (0.61-1.07)	0.135
Duration since diagnosed with HIV	0.83 (0.73-0.95)	0.008	0.95 (0.68-1.35)	0.791
Duration since initiation on ART	0.83 (0.72-0.955)	0.009	0.94 (0.66-1.36)	0.759

ART, antiretroviral therapy; DTG, dolutegravir; HR, hazard ratio; IQR, interquartile range; LPV/r, Lopinavir/ritonavir, ATV/r, Atazanavir/ritonavir; NVP, Nevirapine; EFV, Efavirenz.

## Discussion

Worldwide, HIV remains a public health issue. Despite the introduction of T&T strategies, individuals initiated on ART are not staying in touch with their health care providers as they should be. This is a big problem because it means that they might not be receiving optimal care. Therefore, this study aimed to determine the incidence and predictors LTFU among South Sudanese refugees with HIV receiving care in Adjumani District. In this study, we found the incidence of LTFU among the participants at 5.6 per 100 population and it highlight two significant factors that independently contribute to the risk of LTFU: being male and receiving care from HC IV. Being male and receiving care from HC IV were 2.6 times and three times at risk of LTFU, respectively, among the participants.

The incidence of LTFU was 5.6 per 100. This finding is consistent with a study done among individuals living with HIV in south Ethiopia [14]. However, other studies report higher incidence of LTFU, for example, a study done in Masaka, Uganda reports an incidence rate of 7.5 per 100 [9]. Zambia reports an incidence of 14.1 per 100 after 5 years of follow-up [15], a study from northeast Ethiopia reports an incidence of 8.9 per 100 [16], another study from northwest Ethiopia reported an incidence rate of 9.7 per 100 [17], one study in Kigali, Rwanda reports an incidence of 9.4 per 100 [18], and a study done in Tanzania reports a much higher incidence rate of 42% among adolescents [19]. Conversely, other studies report lower incidence rates of LTFU [11,20]. There is considerable variation in the incidence of LTFU across different studies and regions. Factors such as geographical location, health care infrastructure, socio-economic conditions, and demographic characteristics (e.g. age) can influence LTFU rates. Higher rates of LTFU suggest potential challenges in maintaining continuity of care and adherence to HIV treatment. Lower rates may indicate more effective retention strategies or better health care systems.

Males were 2.6 times more likely to experience LTFU than females. This finding is consistence with finding from a done in sub-Saharan Africa [21] and northwest Ethiopia [17]. This finding relates with existing literature that suggests than females. Possible explanations include socio-cultural factors influencing health care utilization patterns, such as men being less likely to seek health care due to traditional gender roles or societal expectations. Interventions aimed at improving male engagement in health care services, such as targeted health education campaigns and flexible clinic hours, could potentially mitigate this disparity.

Furthermore, individuals receiving care from HC IV were three times more likely to be lost to follow-up than those receiving care from HC III. HCs IV typically serve larger populations and may face challenges related to staffing, resource constraints, or geographical accessibility, all of which could contribute to higher rates of LTFU. This was also reported by a study done in Wakiso District, Uganda [11]. Strategies to address these issues may include strengthening health care infrastructure at these centers, enhancing training and support for health care workers, and improving transportation options for patients.

### Strength and limitation of the study

The strengths of our study include its robust statistical analysis, which controlled for potential confounding variables. However, several limitations should also be considered. First, the study was conducted in a specific geographical area with its unique health care system and patient demographics, limiting generalizability to other settings. Second, the reliance on medical records for data collection may have introduced biases due to incomplete documentation or data entry errors.

## Conclusion

The incidence of LTFU among South Sudanese refugees with HIV receiving care in public facilities in Uganda was high and is associated with being male and receiving care from HC IV. Early diagnosis, routine use of patient address locator forms, and improved quality of HIV care at HC IV may reduce LTFU among refugees with HIV in Uganda and similar settings.

## Declarations of competing interest

The authors have no competing interests to declare.
